# Equations for predicting DXA-measured visceral adipose tissue mass based on BMI or weight in adults

**DOI:** 10.1186/s12944-022-01652-8

**Published:** 2022-05-16

**Authors:** Xuan Song, Hongxia Wu, Wenhua Zhang, Bei Wang, Hongjun Sun

**Affiliations:** 1 Department of Medical Ultrasound, Shandong Provincial Qianfoshan Hospital, Cheeloo College of Medicine, Shandong University, Shandong Medicine and Health Key Laboratory of Abdominal Medical Imaging, Jinan, Shandong China; 2 Department of Nursing, The First Affiliated Hospital of Shandong First Medical University & Shandong Provincial Qianfoshan Hospital, Jinan, Shandong China; 3 Department of Medical Ultrasound, The First Affiliated Hospital of Shandong First Medical University & Shandong Provincial Qianfoshan Hospital, Jinan, Shandong China

**Keywords:** Body mass index, Weight, Visceral adipose tissue, Equation

## Abstract

**Background:**

Obesity, especially presenting with excessive amounts of visceral adipose tissue (VAT), is strongly associated with insulin resistance (IR), atherosclerosis, metabolic syndrome, and cardiovascular diseases (CVDs).

**Aims:**

To construct a predication equation for estimating VAT mass using anthropometric parameters and validate the models with a validation group.

**Methods:**

Five hundred fifteen subjects (366 for the derivation group and 149 for the validation group) were enrolled in the study. The anthropometric parameters, blood lipid profile, and VAT mass were accessed from medical records. Stepwise regression was applied to develop prediction models based on the dual X–ray absorptiometry (DXA)-measured VAT mass in the derivation group. Bland–Altman plots and correlation analysis were performed to validate the agreements in the validation group. The performance of the prediction equations was evaluated with the Hosmer–Lemeshow test and area under the curve (AUC).

**Results:**

Model 1, which included age, sex, body mass index (BMI), triglyceride (TG), high-density lipoprotein (HDL), and the grade of hepatic steatosis, had a variance of 70%, and model 2, which included age, sex, weight, height, TG, HDL, and the grade of hepatic steatosis, had a variance of 74%. The VAT mass measured by DXA was correlated with age, sex, height, weight, BMI, TG, HDL, and grade of hepatic steatosis. In the validation group, the VAT mass calculated by the prediction equations was strongly correlated with the DXA–VAT mass (*r* = 0.870, *r* = 0.875, respectively). The AUC, sensitivity, and specificity of the two prediction equations were not significantly different (both *P =* 0.933).

**Conclusion:**

The study suggests that prediction equations including age, sex, height, BMI, weight, TG, HDL, and the grade of hepatic steatosis could be useful tools for predicting VAT mass when DXA is not available.

**Supplementary Information:**

The online version contains supplementary material available at 10.1186/s12944-022-01652-8.

## Introduction

Obesity is a global public health issue involving excessive adipose tissue deposition, and its prevalence has surged over the last half century and continues to increase worldwide. Obesity is a heterogeneous disorder, which means that obese individuals may have substantial differences in body fat deposition and health risk levels despite similar body weight [1, 2]. Surging evidence suggests that the regional distribution rather than the total amount of adipose tissue is more important for the morbidity and mortality of metabolic diseases [1, 2]. Fat that is mainly present in subcutaneous areas is defined as subcutaneous adipose tissue (SAT), and fat mainly deposits in the mesentery and omentum is named visceral adipose tissue (VAT). There are substantial differences between SAT and VAT in terms of cellular, molecular, physiological and metabolic aspects [3]. Current studies show that VAT is likely a stronger indicator than hepatic steatosis, insulin resistance (IR), dyslipidemia, metabolic syndrome and cardiovascular risk.

Several factors, such as age, sex, race and genes, might be involved in the regional distribution of adipose tissue [1]. Males have significantly higher VAT mass than females among both young and aged individuals and increases with age in both males and females [4, 5]. The ratios of VAT mass to total body mass were approximately 10–20% in males and 5–8% in females [6]. Body mass index (BMI), weight or waist circumference (WC) is mostly used as an alternative measurement for the obesity of individuals in clinical practice due to its convenience and simplicity. However, the surrogate measurements of obesity are not highly correlated with directly measured fat mass, SAT, or VAT, which suggests that they do not sufficiently reflect the variation in total body fat and VAT because fat is not evenly distributed. WC might crudely estimate the degree of abdominal obesity; however, distinctions cannot be made between VAT and SAT when WC decreases with weight loss [1]. Magnetic resonance imaging (MRI) and computed tomography (CT) have been shown to evaluate body composition in total and local regions, allowing the measurement of body fat, muscles, and bones. Recently, dual energy X–ray absorptiometry (DXA) has gradually become the preferred method for assessing the body fat mass, lean mass, and bone mineral content of total and specific anatomical regions. Estimation of VAT can also be done based on the measurements of the abdominal region. Although the method for measuring VAT by DXA is different from that by CT or MRI, DXA has been proven to be strongly correlated with MRI and CT [7–11]. The association of DXA–VAT with IR, cardiovascular factors, and blood lipids was comparable to that of CT–VAT [12, 13]. Compared with MRI or CT, DXA has higher efficiency and lower effective radiation exposure. However, DXA has limitations for healthy screening due to its high cost, lack of convenience, and feasibility issues; hence, finding an indirect simple prediction equation to estimate VAT mass is necessary. In this study, we attempted to construct prediction models based on age, sex, weight, height, BMI, high-density lipoprotein (HDL), triglyceride (TG), and grading of hepatic steatosis with a derivation group and validated the model with a validation group.

## Materials and methods

### Subjects

A total of 515 subjects older than 20 years who underwent whole-body DXA and abdominal ultrasound from November 2019 to August 2021 at Shandong University Affiliated Qianfoshan Hospital were included. Participants with alcohol intake > 20 g per day, autoimmune hepatic disease, viral hepatitis, systematic disease, serious chronic illness, or malignant disease or who used medicine that might interfere with adipose infiltration were excluded. The ethics committee of Shandong University Affiliated Qianfoshan Hospital approved this retrospective study. (NO. S1181).

The clinical data of all subjects were obtained from their medical documentation, including age; sex; height; weight; lipid profiles; systolic blood pressure (SBP) and diastolic blood pressure (DBP); history of diabetes mellitus (DM) and hypertension. VAT mass was measured by a DXA Lunar scanner (GE Healthcare, Madison, WI, USA), and the measured values were collected from the DXA dataset.

All retrospective images were obtained with LOGIQ® E9 (GE Healthcare with Encore version 17.0, Waukesha, WI, USA) and collected from the ultrasound dataset. Hepatic steatosis is usually classified as normal, mild, moderate, or severe by ultrasound based on liver echo pattern, liver–kidney contrast, and the visual appearance of intrahepatic vessels and diaphragm. All participants were divided into four groups based on a previous study [14]. Group 1 (normal): normal echogenicity; Group 2 (mild): slightly brighter liver echogenicity with well-seen liver vessels and diaphragm; Group 3 (moderate): moderately brighter liver echogenicity with slightly affected visualization of the liver vessels and diaphragm; Group 4 (severe): markedly brighter liver echogenicity with poor or no visualization of the liver vessels, diaphragm, and right lobe posterior segment of the liver.

Three experienced sonographers with more than 5 years of experience blinded to the subjects’ clinical details independently reviewed the ultrasound images to evaluate the interobserver reliability. One week later, 150 subjects were randomly selected, and the images were reviewed by one sonographer to evaluate the intraobserver reliability.

## Statistical analyses

All statistical analyses were performed with SPSS 24.0 (SPSS Inc., Chicago, IL, USA) and MedCalc 19.0.4 (MedCalc Software Inc., Ostend, Belgium) software. A *P* value less than 0.05 was considered statistically significant. The Kolmogorov–Smirnov test was used to evaluate the normal distribution of the data. Categorical and continuous variables were expressed as numbers with percentages and medians ± interquartile ranges (IQRs), respectively. Differences in categorical variables were tested using the chi–square test. Inter- and intraobserver agreements were determined by the intraclass correlation coefficients (ICCs) with the two-way random-effects model. An ICC of greater than 0.80 was considered to be excellent. A 95% confidence interval (CI) was calculated for each ICC. The differences in anthropometric parameters, blood lipid profiles and VAT mass between the two groups were evaluated with the Mann–Whitney U test. The Kruskal–Wallis test with adjustment by Bonferroni correction was performed to determine the differences in VAT mass among groups with different grades of hepatic steatosis. The correlation coefficients between the VAT mass and included parameters were evaluated with Spearman’s correlation analysis. Multiple linear stepwise regression analysis was used to develop prediction models with DXA–VAT mass as a dependent variable for males and females both together and separately. The correlations between DXA–VAT mass and the predicted VAT mass were assessed using Spearman’s correlation. Bland–Altman plots were drawn to illustrate the agreement of the prediction models. Separate receiver operating characteristic (ROC) curves were constructed for DXA–VAT masses less than and greater than1280g. The sensitivity, specificity, and area under the curve (AUC) were calculated to determine the performance of the prediction models. The differences in the AUC between the two models were compared using DeLong’s test. The Hosmer–Lemeshow test was performed to assess the calibration of the prediction models.

## Results

### Study demographics and anthropometric characteristics

A total of 515 subjects (262 males and 253 females) were recruited for our study. The median age was 58 years (IQR = 50–65 years) for all participants, 57 years (IQR = 49–64 years) for males and 60 years (IQR = 52–66 years) for females (Suppl. Table 1).

Subjects were randomly assigned to the derivation group (*n* = 366) or validation group (*n* = 149). The clinical anthropometric and metabolic parameters of the derivation group and validation group are shown in Table 1. The differences between the two groups in age, sex, weight, height, BMI, ALT, TC, TG, HDL, LDL, VAT, DM% or HBP% had no statistical significance. The fasting plasma glucose (FPG) and AST levels were significantly different between the two groups (Table 1).

**Table 1:** Basic anthropometric and metabolic parameters

	Total	Derivation group	Validation group	*P*-value
Number	515	366	149	
Sex				0.259
Male	262 (50.9%)	192 (52.5%)	70 (47.0%)	
Femal	253 (49.1%)	174 (47.5%)	79 (53.0%)	
Age (years)	58 (50–65)	58 (51–65)	58 (50–64)	0.338
HR	79 (72–88)	79 (72–88)	80 (73–88)	0.894
SBP	133 (122–147)	133.5 (122–147)	133 (121–148)	0.673
DBP	79 (72–87)	79 (71–87)	80 (72–88)	0.417
Grade of hepatic steatosis				0.237
Normal	216 (41.9%)	156 (42.6%)	60 (40.3%)	
Mild	176 (34.2%)	127 (34.7%)	49 (32.9%)	
Moderate	100 (19.4%)	71 (19.4%)	29 (19.5%)	
Severe	23 (4.5%)	12 (3.3%)	11 (7.4%)	
Height(cm)	167 (160–173)	167 (160–174)	167 (160–172)	0.122
Weight(kg)	69.4 (61.0–81.7)	70.8 (60.6–81)	68 (61.5–83.4)	0.986
BMI(kg/m2)	25.3 (22.9–28.2)	25.2 (22.8–28.2)	25.4 (23.3–28.1)	0.568
VAT	1283 (843–1837)	1252.5 (836.5–1840.75)	1357 (857.5–1835.5)	0.253
FPG	6.3 (5.0–8.71)	6.5 (5.0–9.0)	6.0 (4.8–8.6)	0.007
ALT	15.8 (11.5–23.8)	15.8 (11.5–24.1)	15.7 (11.9–22.4)	0.200
AST	17.0 (13.9–21.7)	17.0 (13.7–21.8)	16.9 (14.0–21.0)	0.009
TC	4.5 (3.7–5.3)	4.5 (3.7–5.3)	4.7 (3.8–5.3)	0.316
TG	1.3 (0.9–1.9)	1.3 (0.9–1.9)	1.4 (0.9–1.9)	0.477
HDL	1.1 (1.0–1.3)	1.1 (1.0–1.4)	1.1 (0.9–1.3)	0.143
LDL	2.6 (2.0–3.2)	2.6 (2.0–3.2)	2.8 (1.9–3.3)	0.709
DM (n/%)	343/66.6%	251/68.6%	92/61.7%	0.136
Hypertension (n/%)	234/45.4%	160/43.7%	74/49.7%	0.219

It shows mean median and interquartile range (IQR). *Abbreviations: ALT* alanine aminotransferase, *AST* aspartate aminotransferase, *BMI* body mass index, *DBP* diastolic blood pressure, *DM* Diabetes mellitus, *FPG* fasting plasma glucose, *HBP* high blood pressure, *HDL* high density lipoprotein, *HR* heart rate, *SBP* systolic blood pressure, *TC* total cholesterol, *TG* triglyceride, *VAT* visceral adipose tissue

### Observer reliability

The inter- and intraobserver agreements were excellent. The interobserver correlation coefficient for the three sonographers was 0.905 (95% CI: 0.891–0.918). The intraobserver correlation coefficient for the sonographer was 0.921 (95% CI: 0.891–0.943).

### Correlation between VAT mass and clinical characteristics

**Fig. 1:**
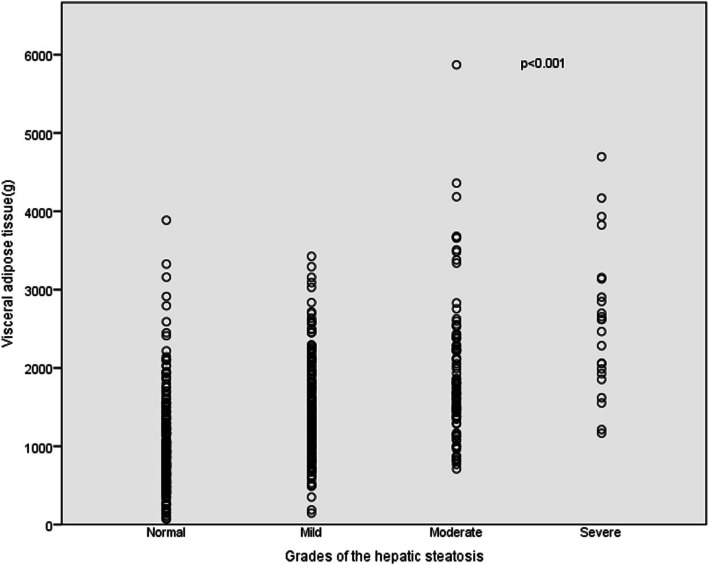
Distribution of visceral adipose tissue (VAT) mass in subjects with different grading of hepatic steatosis

The DXA–VAT mass and the grade of hepatic steatosis measured by ultrasound was moderately correlated (*r* = 0.527, *P* < 0.001, Table 2); that is, the VAT mass gradually increased as the severity of hepatic steatosis increased. Subjects in Group 3 and Group 4 had a larger VAT mass than those in the normal and mild groups, while subjects in Group 2 had a larger VAT mass than those in Group 1 (all *P* < 0.001). However, no statistical significance was found in terms of VAT mass between the moderate and severe groups (*P* = 0.121) (Fig. 1). The correlations between VAT mass and age, height, weight, BMI, TG, and HDL are shown in Table 2.

**Table 2:** The statistical correlation with DXA–VAT mass

Parameters	*r*	*P*-value
Age	0.007	0.877
Height	0.456	< 0.001
Weight	0.800	< 0.001
BMI	0.746	< 0.001
TG	0.371	< 0.001
HDL	−0.374	< 0.001
Grade of hepatic steatosis	0.527	< 0.001

*Abbreviations: BMI* body mass index, *HDL* high density lipoprotein, *TG* triglyceride

### Regression model construction and validation

In the derivation group, the final stepwise linear regression model included all the tested variables. Linear regression model 1 for estimating VAT mass included age, sex, BMI, HDL, TG, and the grade of hepatic steatosis (*F* = 143.074, *P* < 0.001). Linear regression model 2 for estimating VAT mass included age, sex, height, weight, HDL, TG, and the grade of hepatic steatosis (*F* = 156.734, *P* < 0.001). The final prediction equations generated using multiple stepwise methods for the derivation group are shown in Table 3. The detailed regression equations for each model in the derivation group are shown in Suppl. Table 2 and Suppl. Table 3. The subgroup models for males and females were also constructed (Suppl. Table 4). The variances of subgroup model 1 for males and females were 0.683 and 0.681, respectively, which were slightly lower than the variance of model 1 for both sexes. The variances of subgroup model 2 for males and females were 0.739 and 0.706, respectively, which were also slightly lower than those of model 2 for both sexes.

**Table 3:** Regression models for predicting VAT mass in the derivation group

Models	U-β	S-β	*P*-value	*R*^2^	Adjusted *R*^2^	SEE	D-W
**Model 1**				0.705	0.700	447.399	2.122
Constant	− 2117.622		< 0.001				
BMI	121.839	0.674	< 0.001				
Sex	− 479.438	−0.293	< 0.001				
Age	16.845	0.241	< 0.001				
Grade of hepatic steatosis	125.787	0.131	< 0.001				
HDL	− 157.300	−0.086	0.003				
TG	39.668	0.076	0.013				
**Model 2**				0.754	0.749	409.229	2.014
Constant	1901.611		0.026				
Weight	45.290	0.900	< 0.001				
Age	19.325	0.277	< 0.001				
HDL	−157.105	−0.086	0.001				
Grade of hepatic steatosis	91.653	0.096	0.005				
Height	−26.518	−0.268	< 0.001				
Sex	− 328.137	−0.201	< 0.001				
TG	33.212	0.063	0.023				

*Abbreviations: BMI* body mass index, *HDL* high density lipoprotein, *TG* triglyceride

**Fig. 2:**
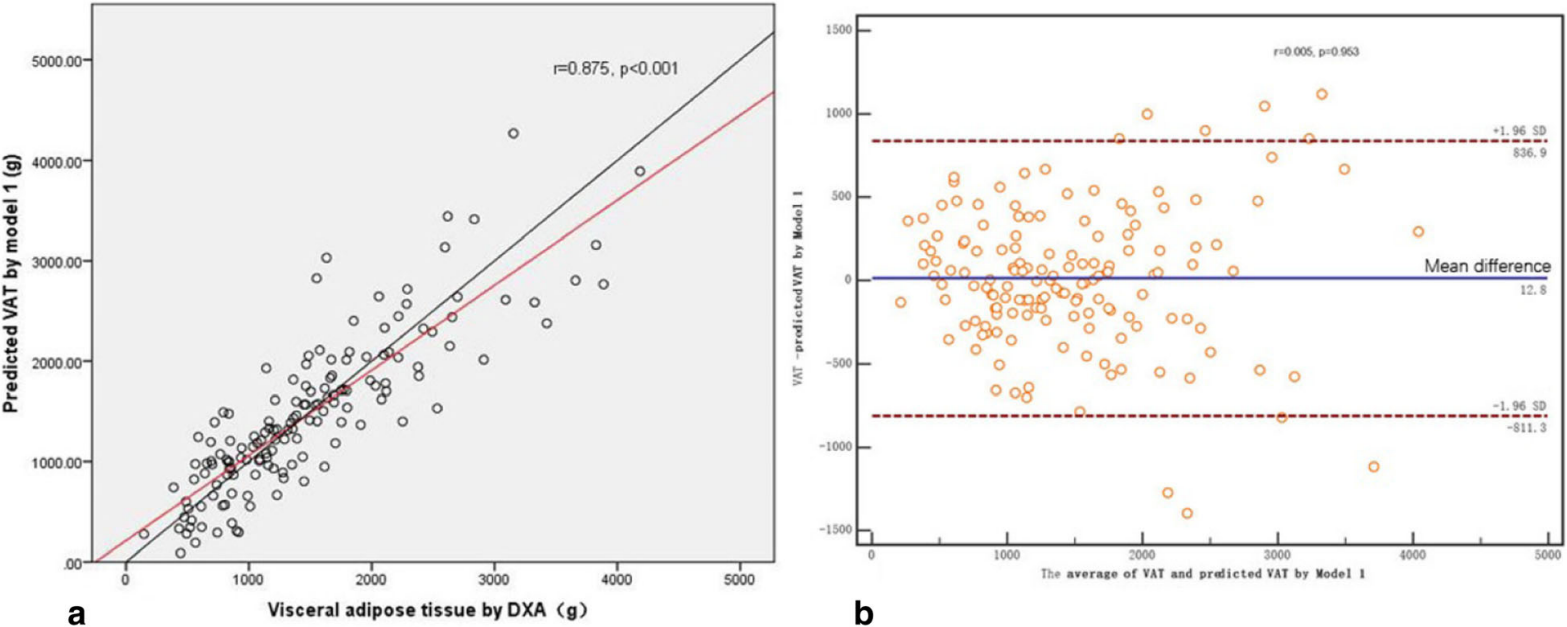
**a** Correlation between DXA–VAT mass and predicted VAT by Model 1. **b** Bland–Altman plots for DXA–VAT mass and predicted VAT by Model 1 with 95% limits of agreement. The middle line indicates the mean difference between DXA measured VAT and predicted VAT. The red dotted lines represent the limits of agreement (LoA)

**Fig. 3:**
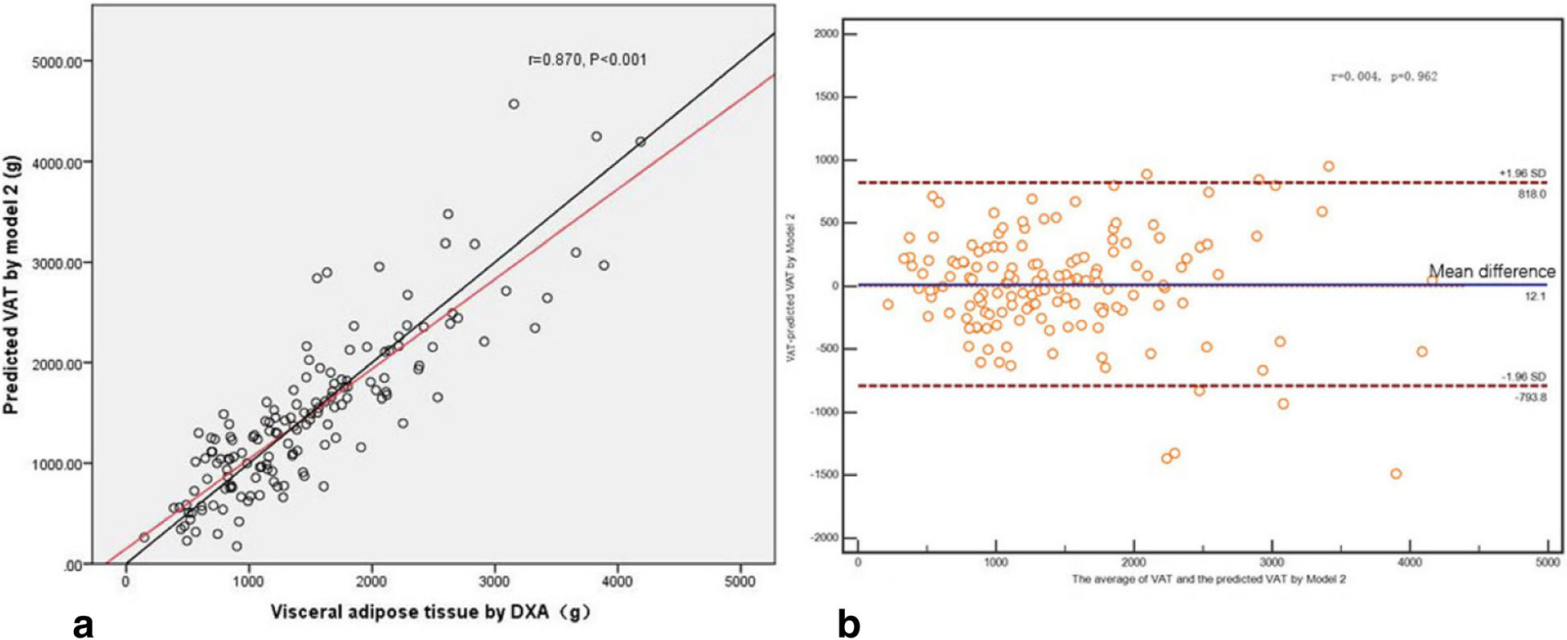
**a** Correlation between DXA–VAT mass and predicted VAT by Model 2. **b** Bland–Altman plots for DXA–VAT mass and predicted VAT by Model 2 with 95% limits of agreement. The middle line indicates the mean difference between DXA measured VAT and predicted VAT. The red dotted lines represent limits of agreement (LoA)

In the validation group, DXA–VAT mass was significantly correlated with the predicted VAT mass calculated from model 1 (*r* = 0.875, *P* < 0.001, Fig. 2a) and that calculated from model 2 (*r* = 0.870, *P* < 0.001, Fig. 3a). There was a mean bias of 12.8 between the DXA–VAT mass and predicted VAT mass estimated by model 1 (95% IC: − 55.266–80.876, Fig. 2b), and the proportional bias was not significant (*r* = 0.005, *P* = 0.953). The mean bias between the DXA–VAT mass and predicted VAT mass by model 2 was 12.1 (95% IC: − 54.479–78.646, Fig. 3b), and the proportional bias was also not statistically significant (*r* = 0.004, *P* = 0.962).

### Prediction model performance

ROC curves were drawn for DXA–VAT < 1280 g (*n* = 70) and VAT ≥1280 g (*n* = 79). The AUC, sensitivity, and specificity of the two prediction equations for estimating VAT are shown in Table 4. The AUCs of the two equations revealed no statistical significance (*P* = 0.933). The Hosmer–Lemeshow test showed that the *P* values of model 1 and model 2 were 0.696 and 0.683, respectively.

**Table 4:** The AUC of ROC,sensitivity, and specificity of two equations in the validation group

Model	Model	Sensitivity	Specificity	*p*
1	0.952 (0.904-0.980)	91.43% (82.8%-96.8%)	88.61%(79.5%-94.7%)	0.933
2	0.951(0.903-0.980)	92.86%(84.1%-97.6%)	86.08%(76.5%-92.8%)	

## Discussion

In the present study, prediction models were constructed to estimate the DXA–VAT. Both constructed prediction models had favorable variances. Although linear regression model 2 better explained the variance than model 1, the AUCs were not significantly different between the two models.

First, the general correlations between VAT mass and included parameters were assessed. The VAT mass was strongly correlated with weight and BMI, which was consistent with the findings in Spadaccini et al.’s study [15]. In a study by Spadaccini et al., the correlation assessments between VAT mass and different variables suggested that weight and BMI were better independent variables associated with VAT mass. Linear regression in the present study indicated that weight and BMI were the top variables in the equations, while the inclusion of additional variables increased the variance of the regression model over using BMI or weight alone, although either weight or BMI might be useful for estimating VAT mass. This might be because combining weight and height better explains the VAT mass differences between males and females.

VAT mass was not strongly correlated with age in this study, which was similar to the findings in a previous study [15]. However, age was an independent parameter associated with VAT mass after correcting for confounders. Several studies have demonstrated that body fat distribution is associated with age and sex. Excess adipose tissue is stored preferentially in SAT rather than VAT in young and middle-aged individuals [16]. However, VAT increased with age, while SAT showed the opposite trend. Large sample studies on the reference for DXA–VAT mass suggest that males have larger VAT mass than females across the whole age range and that VAT mass basically increases with age in both sexes [5, 17]. In this study, males had significantly higher VAT mass than females. Sex increased the variance by 8% in model 1, which was larger than that in model 2 (1.5%). The height and weight were considerably different between males and females. Therefore, the variance difference between the two models was mostly explained by the larger coefficient of height and weight in model 2.

Abnormal lipid levels combined with excessive visceral fat included an increase in TG, a decrease in HDL cholesterol, and almost unchanged LDL cholesterol [2]. HDL is more sensitive to degradation in visceral obesity subjects and more prone to be clear from the blood [2]. Amato et al. [18] proposed a visceral adiposity index (VAI) according to sex, WC, TG, and HDL, which was corroborated to be a valuable index for reflecting visceral fat function and assessing cardiometabolic risk. In this study, the correlation between VAT mass and HDL was negative, while the correlation between VAT and TG was positive, which was consistent with a previous study [15, 18].

Abdominal ultrasound is widely used as a diagnostic modality to assess hepatic steatosis in the clinic and is recommended as a screening method for metabolic-associated fatty liver disease (MAFLD) [19]. It is possible that the performance of the prediction equations could have been compromised by the inclusion of ultrasound due to its reliability. In this study, the intra- and interobserver reliability of the sonographers was excellent. The sensitivity and specificity of ultrasound have been shown to be relatively considerable compared to those of histology in assessing hepatic steatosis [20–22]. In Hamaguchi et al.’s study [22], the severity of hepatic steatosis reflected visceral fat accumulation in healthy people without excessive alcohol consumption. In this study, the severity of hepatic steatosis was positively correlated with the amount of DXA–VAT mass. In the regression analysis, the grade of hepatic steatosis in the two models developed in this study demonstrated similar variance.

Excess adipose tissue induced VAT and ectopic fat deposition in the liver, muscle and pericardium. Epicardial adipose tissues (EAT), present in the epicardium, could promote higher proinflammatory cytokines and influence the myocardial function and structure of the left atrium (LA) and left ventricle (LV) [23]. HDL is thought to reverse cholesterol transport, reduce cholesterol deposition in blood vessel walls, and prevent atherosclerosis, while the accumulation of LDL promotes atherosclerosis. The lipoprotein (a) [Lp(a)] combining an apolipoprotein(a) [apo(a)] with an apolipoprotein B 100 (apoB100) of LDL was also a predictor for atherosclerosis and CVD [24, 25]. Hence, VAT was associated with ectopic fat deposition, atherosclerosis, and cardiovascular disease (CVD).

Ideally, the limits of agreement of the Bland–Altman plots would be smaller for an equation to be considered more accurate when estimating VAT mass. In this study, the VAT mass estimated by the equations was in good agreement with those measured by DXA in the validation group. The agreements were highly significant, with small mean biases for the two models and no systematic errors in the two equations. Both models had favorable calibration and AUCs, which suggested that their performance was perfect.

## Comparisons with other studies

Previous studies have reported several prediction models that can evaluate VAT based on the measurement of CT or MRI [26–29]. However, there are few studies that predict VAT based on the measurement of DXA [30]. In previous studies, age, sex, BMI, height, or WC were mostly included, which resulted in variances ranging from 40 to 76% [26–29]. Unlike those studies, the blood lipid profile and an abdominal ultrasound were included in the regression models in this study. The total models and subgroup models were constructed using stepwise regression analysis, and two models with the highest variance were proposed. In this study, although the lipid profile was considered, only TG and HDL were included in the models using stepwise regression analysis. The results suggest that TG and HDL are the two main parameters in abnormal lipid metabolism that are associated with excessive VAT. The grade of hepatic steatosis evaluated by ultrasound was also included in the regression models. Correlation analysis demonstrated that the severity of hepatic steatosis measured by ultrasound was positively correlated with the amount of DXA–VAT, which suggests that this parameter might reflect the visceral fat amount to a certain degree in individuals with hepatic steatosis. Weight and BMI were included in the two models because they had a similar degree of association with DXA–VAT. Combining more parameters resulted in higher variance than BMI or weight alone.

## Strengths and limitations

The study offers several strengths. First, this study constructed prediction models to evaluate VAT mass assessed by DXA for the first time. Multiple stepwise regression analysis was used to select the most favorable models. Second, this study included the parameters of the lipid profile and abdominal ultrasound, BMI, or weight in models, which obtained higher variances. Furthermore, Bland–Altman plots, ROC curves and the Hosmer–Lemeshow test were performed to test the performance of the models.

Nevertheless, the clinical data obtained were single-center data from China, and the sample size was limited. Thus, further studies should include large sample sizes to better assess the reliability of the equations. Retrospective data limit the observation of individual changes in VAT mass within a period of time. Longitudinal studies are required to observe whether the prediction equations are suitable for subjects whose VAT mass changes.

## Conclusions

The prediction models constructed in this study are based on several common and easily measured parameters, and these models demonstrated high performance with DXA. This study demonstrates that these prediction equations could be useful and easily applicable tools for predicting VAT mass when DXA is unavailable in the clinic. They could also be used in conditions in which DXA is unavailable for the diagnosis, treatment, and prognosis of visceral obesity–related diseases. This might be of certain clinical value and have several public health implications. These prediction models might also provide new information for future research. This might be a novel area of interest and new point for future studies.

## Supplementary Information

**Additional file 1.**

**Additional file 2.**

**Additional file 3.**

**Additional file 4.**

## Data Availability

The datasets analyzed during the current study available from the corresponding author on reasonable request.

## Abbreviations

ALT: Alanine aminotransferase
AST: Aspartate aminotransferase
AUC: Area under the curve
BMI: Body mass index
CI: Confidence interval
CVD: Cardiovascular disease
DBP: Diastolic blood pressure
DM: Diabetes mellitus
DXA: Dual X–ray absorptiometry
EAT: Epicardial adipose tissue
FPG: Fasting plasma glucose
HDL: High density lipoprotein
ICC: Intraclass correlation coefficient
IQR: Interquartile range
IR: Insulin resistance
LA: Left atrium
LV: Left ventricle
LoA: Limits of agreement
LDL: Low density lipoprotein
MAFLD: Metabolic associated fatty liver disease
ROC: Receiver operating characteristic
SAT: Subcutaneous adipose tissue
SBP: Systolic blood pressure
SD: Standard deviation
TC: Total cholesterol
TG: Triacylglycerol
VAT: Visceral adipose tissue
VAI: Visceral adiposity index
WC: Waist circumference
